# Syphilitic Disease of the Brain

**Published:** 1904-09-03

**Authors:** 


### SYPHILITIC DISEASE OF THE BRAIN.

Now that the public is beginning to realise the great burden entailed upon the community by the increasing numbers of the insane who are inmates of public asylums, it is some satisfaction to recall that in a large percentage of the cases the mental trouble has been due to causes which may rightly be considered as preventable. That being so it is likely that more strenuous efforts will be made in the future to obviate the evil factors. Indulgence in alcohol is one of the chief agencies in the development of mental defects, and here wise statesmanship has already come to the aid of professional opinion in combating the evil. The same may be possible with regard to syphilis, which, in the opinion of Dr. Mott[1] "is by far the most important cause of organic brain-disease in adults and . . . is one of the most weighty intrinsic factors in the production of insanity." From 20 to 25 per cent. of the male adults attending his out-patient department for various diseases, he states, had been certainly affected with syphilis, while a much greater number had suffered from some form of venereal disease, and it has been calculated that about one to two per cent. of persons infected with syphilis suffer from cerebral symptoms exclusive of parasyphilitic affections.

The various forms of brain disease in syphilis may be considered under the headings of: (1) basic meningitis, (2) meningitis of the convexity, (3) cerebro-spinal meningitis, (4) arteritis and neoplastic formations, and (5) encephalitis. Of these the first named is the most common. The inflammatory process frequently commences about the optic chiasma and causes severe headache, which is usually most intense at night or in the early morning. Mental symptoms are severe, and the cases have a close resemblance to general paralysis; hence mistakes in diagnosis are frequent. In syphilitic meningitis of the base, however, affections of the optic nerves and of the nerves supplying the ocular muscles are common, the oculo-motor nerve being the one most frequently involved; but it is the rule for the paralysis to be incomplete, some of the muscles supplied by the oculo-motor nerve retaining their function. The muscle especially likely to be paralysed, and the earliest also to suffer, is the levator palpebræ, and ptosis, therefore, should always raise a suspicion of syphilis. Recurrent attacks of drowsiness, stupor, and coma are marked features in some cases, and many different forms of insanity may be simulated—*e.g.* epilepsy, general paralysis, melancholia, etc.

No hard and fast line can be drawn between syphilitic disease of the base and of the convexity of the brain, and the two lesions may be present at the same time. Headache is the most common and sometimes the only symptom of meningitis of the convexity, and is often extremely severe. Optic neuritis is uncommon. Vomiting, stupor, giddiness, and psychical disturbance may supervene. Gummatous growths on the convexity may give rise to definite localising symptoms, *e.g.* when situated in the motor area. The existence of severe headache with local tenderness, especially if accompanied by a history of syphilis, should lead to a trial of anti-syphilitic remedies.

Cerebro-spinal meningitis, though frequently seen in the post-mortem room, is overlooked clinically, as a rule, on account of predominating brain symptoms. Its manifestions are stiffness in the neck, pains in the back, disturbance of the sphincters, etc.

Syphilitic arteritis affects principally the inner coat of the arteries, and leads to narrowing of the lumen and thrombosis. Headache is a common and early symptom. Transitory attacks of aphasia, monoplegia, or hemiplegia are signs which point to obliterative arteritis, and it is most important that they should not be neglected, for the only hope of doing the patient good is to place him on anti-syphilitic treatment before occlusion of the vessels and softening of the brain have occurred.

[1] *Practitioner*, July.

